# Food and the Principles of Dietetics

**Published:** 1901-05

**Authors:** 


Food and the Principles of Dietetics. By Robert Hutchison, M. D., Edin., M. R. C. P., Assistant Physician to the London Hospital and to the Hospital for Sick Children, Great Ormond street. Octavo, pp. 566. With plates and diagrams. New York: William Wood & Co. 1900.

The contents of this book, the author announces in the preface, were first addressed to the students of the London Hospital in the form of a course of lectures, their gratifying reception leading to their presentation in book form. The medical public is to be congratulated that circumstances led Dr. Hutchison to the publication of these lectures.

The subject of dietetics is one which is daily demanding more and more attention, and yet many of us are too ignorant to intelligently advise our patients as we ought. To a certain extent foods, their digestibility and their relative nutritive value receive some notice in the current text-books on physiology and are perhaps more or less dwelt upon in lectures upon physiology, hygiene, or medicine. Their economic value is seldom if ever touched upon, yet it is of no small importance to the physician to be able to suit his diet list to his patient's purse. What knowledge the doctor has of the proper cooking of food is usually a matter of chance. He has hardly considered it in his province to know how a potato should be boiled.

Here in this volume are gathered together practically all that is at present known of the subject of dietetics, the book representing the results reached by many careful observers in many lands. Nothing has been considered too trivial to receive attention, if it has a practical bearing on the subject. The relative values of foods, food in health, the effect of occupation upon the amount of food required, detailed analyses of all the common foods, and of many not common, —vegetarianism, beverages, including alcohol, the cooking of foods, digestion, infant feeding, and feeding in disease,—are all considered fully, clearly and practically. Completeness, so far as possible, is evident on every page.

It is a book so full of useful information that the reader cannot but pursue it with profit, and yet so simple and delightful is the author's style that even the layman may read it with pleasure.

